# Behavioral Health Flag Use by Race and Ethnicity in a Pediatric Emergency Department

**DOI:** 10.1001/jamanetworkopen.2025.9502

**Published:** 2025-05-09

**Authors:** Danielle Foltz, Gia M. Badolato, Theresa Ryan Schultz, Shilpa J. Patel, Asha S. Payne, Sephora Morrison, Meleah Boyle, Monika K. Goyal

**Affiliations:** 1 Center for Translational Research, Children’s National Hospital, Washington, DC; 2 Department of Pediatrics, George Washington University Hospital, Washington, DC

## Abstract

**Importance:**

Behavioral health flags are intended to serve as safety alerts for staff by identifying patients at risk of aggression but may also be prone to racial bias, leading to inequities in care.

**Objective:**

To investigate racial and ethnic differences in the use of an aggression risk evaluation tool and being labeled as high aggression risk in the electronic health record among youths presenting to a pediatric emergency department.

**Design, Setting, and Participants:**

This cross-sectional study of electronic health record data was conducted at a single-center pediatric emergency department. Visits from patients aged 21 years or younger presenting with mental health–related concerns between January 2020 and December 2022 were included.

**Exposure:**

Patient race and ethnicity (Hispanic, non-Hispanic Black [hereafter, Black], non-Hispanic White [hereafter, White], or other, which includes individuals who identify as American Indian or Alaska Native, Asian, multiple races, or any other race).

**Main Outcomes and Measures:**

The primary outcomes were (1) the use of an aggression risk evaluation tool by a psychiatric social worker, (2) being labeled with a high aggression risk behavioral health flag, or (3) being labeled with a high aggression risk behavioral health flag with a history of violent behavior documented with no other aggression risk behaviors documented. Separate logistic regression models were used to examine association of race and ethnicity with each outcome.

**Results:**

There were 5121 visits for mental health concerns. The majority of patients were female (3198 patients [62.5%]) and publicly insured (3161 patients [61.8%), with a mean (SD) age of 13.8 (2.7) years. With regard to race and ethnicity, 3061 patients (59.8%) were Black, 893 patients (17.4%) were Hispanic, 778 (15.2%) were White, and 389 (7.6%) belonged to other racial and ethnic groups. Most visits (4119 visits [80.4%]) had an aggression risk evaluation tool completed, and 627 (15.2%) were labeled with a high aggression risk behavioral health flag. There were no racial and ethnic differences in use of the aggression risk evaluation tool. Compared with White youths, Black youths were more likely to be labeled as high aggression risk (60 White youths [9.8%] vs 486 Black youths [19.8%]; adjusted odds ratio, 1.71; 95% CI 1.24-2.35). Among patients who had a history of violent behavior and no other aggression risk behaviors documented on the evaluation tool, Black patients had higher odds of being labeled as high aggression risk compared with White patients (72 Black patients [32.4%] vs 3 White patients [10.7%]; odds ratio, 4.00; 95% CI, 1.16-13.69).

**Conclusions and Relevance:**

In this cross-sectional study, Black youths were more likely to be labeled with a high aggression risk behavioral health flag despite similar responses to an aggression risk evaluation tool. Processes to ensure equitable care delivery are critically needed.

## Key Points

**Question:**

Are there racial and ethnic differences in the use of an aggression risk evaluation tool or being labeled as high aggression risk among youths presenting to a pediatric emergency department?

**Findings:**

In this cross-sectional study of 5121 mental health–related emergency department visits, there were no racial and ethnic differences in the use of an aggression risk evaluation tool. However, non-Hispanic Black youths had 4 times greater odds of being flagged as high aggression risk compared with non-Hispanic White youths with similar responses on the tool, a statistically significant difference.

**Meaning:**

These findings suggest that non-Hispanic Black youths are disproportionately flagged as high aggression risk, despite responses on an aggression risk evaluation tool similar to those of White youths; future studies should investigate the underlying factors that may contribute to racial and ethnic differences in being labeled with a high aggression risk behavioral health flag.

## Teaser

This cross-sectional study investigates racial and ethnic differences in the use of an aggression risk evaluation tool in a pediatric emergency department (ED).

## Introduction

It is estimated that 1 in 5 youths have a mental health condition.^1^ Since 2016, pediatric emergency department (ED) visits for suicide attempts, suicidal ideation, and self-injury have almost tripled,^2^ with a 20% increase between 2019 and 2022.^2^ Children from minoritized racial and ethnic groups are less likely than non-Hispanic White children to have access to mental health care,^3^ which may be a contributing factor for why ED visits are increasing at a faster rate for non-Hispanic Black children compared with non-Hispanic White children.^4,5^

Historically, Black children have been subjected to interpersonal, institutional, and structural racism that negatively impacts their health care.^6^ Black patients are often overdiagnosed, misdiagnosed, or underidentified as having neurodevelopment disorders, such as attention-deficit/hyperactivity disorder, learning disorders, and autism spectrum disorders, while also being undertreated for these disorders.^4,7^ Structural racism and bias could be factors associated with differential treatment of Black youths in the health system. Prejudices like adultification and anger bias are examples of misperceptions of Black youths as being older and more threatening,^8^ which could result in harsher treatment of Black youths, especially as it relates to management of mental health conditions. In the ED, Black children with mental health conditions receive harsher treatments.^9^ For instance, Black children are more likely to be physically or chemically restrained compared with their White peers.^10,11^ The harsher treatment of Black children in the diagnosis and management of mental health conditions is part of a negative cycle perpetuated by structural racism. For example, harsh treatments can contribute to existing trauma and adverse childhood experiences that later reinforce the need for emergency care, and being restrained could reinforce bias against the patient within the ED, leading to more differential treatment.^8^

As approximately three-quarters of ED nurses and one-quarter of ED physicians have experienced physical assault at work during their careers, electronic health record (EHR)–embedded behavioral health flags have been implemented to alert ED staff to the potential risk of violent or harmful encounters.^12,13^ However, the use of behavioral health flags to prevent violence in the ED is not evidenced based and may be a mechanism for racial bias, thus negatively impacting patient care. Recent studies in the adult population have identified racial and ethnic inequities in behavioral health flag use, but no studies to date have examined inequities in the application of behavioral health flags in the pediatric population.^13,14^

The objective of this study was to investigate racial and ethnic differences in (1) the use of an aggression risk evaluation tool and (2) being labeled with a high aggression risk behavioral health flag in the EHR. The aggression risk evaluation tool is completed by a psychiatric social worker and is used to inform the determination of a low, moderate, or high risk aggression risk flag in the EHR. We hypothesized that youths from minoritized racial and ethnic groups would have a higher likelihood of being labeled with a high aggression risk behavioral health flag.

## Methods

### Study Design and Population

We performed a cross-sectional study of ED visits to a single urban pediatric ED with an annual census of 90 000 visits from January 2020 through December 2022 using EHR data. ED visits of patients 21 years old and younger with mental health concerns, classified as visits with a psychiatry social worker evaluation, were included in this study. Patients were evaluated by a psychiatry social worker if they presented with a mental health concern and met 1 of the following criteria: screening for high risk for suicide, presenting with an application for emergency hospitalization status, suicidal or homicidal ideation, auditory or visual hallucinations, or the doctor requested a psychiatry social work evaluation on the basis of another safety concern. We excluded visits for patients who were critically ill (eg, triaged at the highest acuity level of Emergency Severity Index [ESI] 1), those who were older than 21 years, or if patient race and ethnicity were missing from the EHR. This study was approved by the hospital institutional review board, which granted a waiver of informed consent because the study posed minimal risk to participants and used deidentified data from existing medical records, in accordance with 45 CFR §46. We followed the Strengthening the Reporting of Observational Studies in Epidemiology (STROBE) reporting guidelines for cross-sectional studies.

### Outcomes

This study had 3 outcomes. The first outcome was the use of an aggression risk evaluation tool. The second outcome was being labeled with a high aggression risk behavioral health flag in the EHR. The third outcome examined a subpopulation of patients who were documented as having a history of violent behavior but had no other aggressive behaviors documented in the aggression risk evaluation tool. For this outcome, we compared the presence of high aggression risk behavioral health flags in patients who were documented as having a history of violent behavior but had no other aggressive behaviors documented.

**Figure 1.**
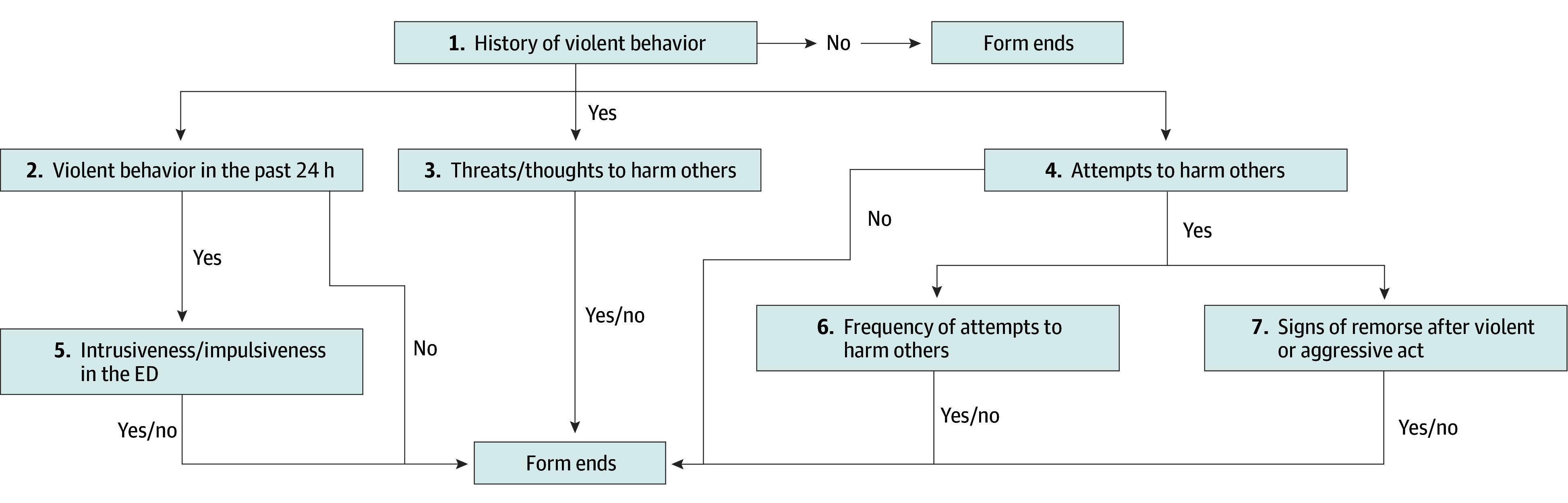
Aggression Risk Evaluation Tool Questions and Branching Logic ED indicates emergency department.

The Brief Rating of Aggression by Children and Adolescents^15^ is a tool validated for use in the pediatric emergency department. The aggression risk evaluation tool is adapted from the Brief Rating of Aggression by Children and Adolescents and was implemented in the ED in 2019 to aid psychiatry social workers in their evaluation for aggression risk among patients who present with mental health crises. This tool includes 8 items: (1) history of violent behavior (yes or no), (2) violent behavior in the past 24 hours (yes or no); (3) threats or thoughts to harm others (yes or no), (4) attempts to harm others (yes or no), (5) intrusiveness or impulsiveness in the ED (yes or no), (6) frequency of attempts to harm others (never, occasional, or often), (7) signs of remorse after a violent or aggressive act (yes or no), and (8) comments. The tool is embedded in an EHR form and completed by the psychiatry social worker. The answers are based on all available information at the time of evaluation and information provided by the patient, their caregivers, or law enforcement (in cases where a child is brought to the ED under emergency hospitalization status). The form has branching logic; if there is no report of history of violent behavior, the form will end. There are no options to add more detailed responses for questions 1 to 7; however, question 8 is a nonrequired free-text option (Figure 1).

The tool does not automatically assign a level of risk on the basis of the answers to the questions. Rather, the psychiatric social worker completes the tool and uses the answers to assign risk and guide placement of a behavioral health flag as low risk, moderate risk, or high risk of aggression. The behavioral health flag is placed on the tracking board on the day of the ED visit. However, if a patient is labeled with a high aggression risk behavioral health flag, an alert will appear in the patient’s EHR at every subsequent ED visit within the health system. This alert will remain in the chart even if the patient is reassessed as low or moderate aggression risk at a subsequent visit.

For analysis, use of an aggression risk evaluation tool was categorized as yes or no if the form was present in the chart and the first question history of violent behavior had a documented response. The presence of a behavioral health flag was categorized as low or moderate aggression risk or high aggression risk. History of violent behavior was summarized as yes or no according to the documented response.

### Measures

The primary exposure variable was the patient’s race and ethnicity. Race and ethnicity are social constructs and can serve as a proxy for measuring the impact of racism on care delivery.^16^ This variable was extracted from the EHR. Race and ethnicity are self-reported by the patient or their caregiver and then entered into the EHR by registration staff. We categorized race and ethnicity as Hispanic, non-Hispanic Black (hereafter, Black), non-Hispanic White (hereafter, White), and non-Hispanic other. Owing to small sample sizes preventing meaningful comparisons, patients identified as American Indian, Alaskan Native, Asian, multiple races, or any other race were collapsed into the other category.^16^ The following demographic and visit variables were also extracted from the EHR as potential covariables: age in years, sex, insurance status, ESI score, and ED disposition.

### Statistical Analysis

We summarized patient and visit characteristic data using descriptive statistics. We calculated the rates of use of the aggression risk evaluation tool, presence of the high aggression risk behavioral health flag, and a yes documented for history of violent behavior without documentation of other aggressive behaviors yet labeled with a high aggression risk behavioral health flag. We developed separate logistic regression models to examine the association between race and ethnicity and each of the aforementioned outcomes. Acknowledging race and ethnicity as social constructs and because minoritized populations often experience bias in care delivery, White was chosen as the referent category. We a priori adjusted our models for outcomes 1 and 2 for the following covariables: age in years, patient sex, and insurance status. The logistic regression model for outcome 3 was left unadjusted because of sample size. Adjusted odds ratios (aORs) were calculated, and statistical significance was defined as 2-sided *P* < .05. All analyses were performed using SAS statistical software version 9.4 (SAS Institute).

## Results

**Figure 2.**
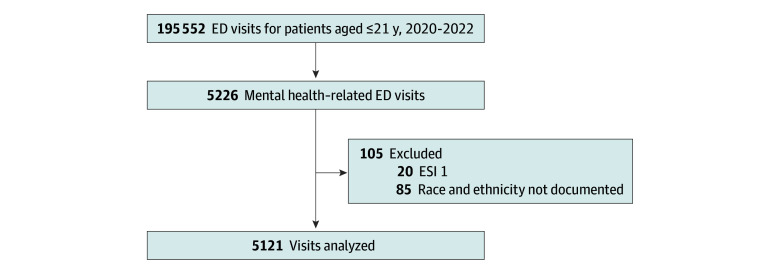
Patient Enrollment Flowchart ED indicates emergency department; ESI, Emergency Severity Index.

There were 5226 mental-health related visits during the study period of which 5121 visits (representing 3761 unique patients) met inclusion criteria. Patients were excluded if they were critically ill, triaged as ESI 1 (20 patients), or if race and ethnicity were missing (85 patients) (Figure 2). The majority of visits were made by patients who were female (3198 patients [62.5%]), with a mean (SD) age of 13.8 (2.7) years. With regard to race and ethnicity, 3061 patients (59.8%) were Black, 893 patients (17.4%) were Hispanic, 778 (15.2%) were White, and 389 (7.6%) were other races (Table 1).

**Table 1.** Patient Demographic Characteristics by Race and Ethnicity

Characteristic	Visits, No. (%)				
	Total (N = 5121)	Hispanic (n = 893 [17.4%])	Non-Hispanic Black (n = 3061 [59.8%])	Non-Hispanic White (n = 778 [15.2%])	Non-Hispanic other (n = 389 [7.6%])^a^
Age, mean (SD), y	13.8 (2.7)	14.0 (2.4)	13.9 (2.8)	13.5 (2.8)	13.9 (2.9)
Sex					
Female	3198 (62.5)	625 (70.0)	1868 (61.0)	468 (60.2)	237 (60.9)
Male	1920 (37.5)	268 (30.0)	1193 (39.0)	307 (39.5)	152 (39.1)
Insurance status					
Public	3161 (61.8)	660 (73.9)	2207 (72.1)	115 (14.8)	179 (46.0)
Private	1758 (34.3)	165 (18.5)	724 (24.4)	653 (83.9)	198 (50.9)
Self-pay	195 (3.8)	67 (7.5)	107 (3.5)	10 (1.3)	11 (2.8)
Not documented	7 (0.1)	NA^b^	5 (0.2)	NA^b^	NA^b^
Emergency Severity Index score					
2	1969 (38.4)	386 (43.2)	1086 (35.5)	333 (42.8)	164 (42.1)
3	3024 (59.1)	488 (54.7)	1874 (61.2)	442 (56.8)	220 (56.6)
4 and 5	128 (2.5)	19 (2.1)	101 (3.3)	NA^b^	5 (1.3)
Dispositioned home	3289 (64.2)	549 (61.5)	2065 (67.5)	444 (57.1)	231 (59.4)

Abbreviation: NA, not available.

^a^ Includes individuals who identify as American Indian or Alaskan Native, Asian, multiple races, or any other race.

^b^ Data are suppressed because cell size is less than 5.

Most visits with a psychiatry social work evaluation had aggression risk evaluation tool used (4119 patients [80.4%]). There were no differences in the use of the tool by patient race and ethnicity (Table 2). Of the visits with a completed evaluation, the majority were labeled with a low aggression risk behavioral health flag (3047 patients [74.0%]).

**Table 2.** Association Between Patient Race and Ethnicity and Aggression Risk Evaluation Tool and High Aggression Risk Behavioral Health Flag Presence in the Electronic Health Record

Race and ethnicity	Aggression risk evaluation tool			High aggression risk flag			Yes to history of violence with no other aggressive behaviors documented		
	Total eligible patients, No. (%)	Used the tool		Total patients eligible, No. (%)	Labeled with a high aggression risk behavioral health flag		Total patients eligible, No. (%)	Labeled with a high aggression risk behavioral health flag	
		Patients, No. (%)^a^	aOR (95% CI)^b^		Patients, No. (%)^a^	aOR (95% CI)^b^		Patients, No. (%)^a^	OR (95% CI)
Hispanic	886 (17.4)	724 (81.7)	0.96 (0.74-1.26)	724 (17.6)	34 (4.7)^c^	0.35 (0.22-0.56)^c^	27 (9.0)	0	NA^d^
Non-Hispanic Black	3038 (59.8)	2459 (80.9)	0.94 (0.75-1.17)	2459 (59.7)	486 (19.8)^c^	1.71 (1.24-2.35)^c^	222 (74.3)	72 (32.4)	4.00 (1.16-13.69)^c^
Non-Hispanic White	771 (15.2)	615 (79.7)	1 [Reference]	615 (14.9)	60 (9.8)	1 [Reference]	28 (9.4)	3 (10.7)	1 [Reference]
Non-Hispanic other^e^	387 (7.6)	321 (83.0)	1.14 (0.82-1.57)	321 (7.8)	47 (14.6)	1.38 (0.90-2.11)	22 (7.4)	4 (18.2)	1.85 (0.37-9.31)

Abbreviations: aOR, adjusted odds ratio; NA, not applicable; OR, odds ratio.

^a^ Row percentages are shown.

^b^ Models are adjusted for age in years, patient sex, and insurance status.

^c^ Denotes significance at *P* < .05.

^d^ Analysis was not powered.

^e^ Includes individuals who identify as American Indian or Alaska Native, Asian, multiple races, or any other race.

Of the patients for whom the aggression risk evaluation tool was used, there were 627 visits (15.2%) where patients were labeled with a high aggression risk behavioral health flag. In the multivariable logistic regression model adjusted for patient age, sex, and insurance, Black youths had higher odds of being labeled with a high aggression risk behavioral health flag compared with White youths (486 Black youths [19.8%] vs 60 White youths [9.8%]; aOR, 1.71; 95% CI, 1.24-2.35), whereas Hispanic youths had lower odds of being labeled with a high aggression risk behavioral health flag than White youths (34 Hispanic youths [4.7%] vs 60 White youths [9.8%]; aOR, 0.35; 95% CI, 0.22-0.56) (Table 2).

Approximately one-third of patients (1468 patients [35.6%]) had a yes response to history of violent behavior. In a subanalysis of 299 patients who had a yes to history of violent behavior but had no other aggressive behaviors documented in the tool, Black youths had higher odds of being labeled with a high aggression risk behavioral health flag in their EHR compared with White youths (72 Black patients [32.4%] vs 3 White patients [10.7%]; OR, 4.00; 95% CI, 1.16-13.69) (Table 2).

## Discussion

In this cross-sectional study of patients who presented to a pediatric ED for mental health concerns, Black patients were labeled with a high aggression risk behavioral health flag more often than White patients. Among a group of youths with similar scores on their aggression risk evaluation tool, Black youths had increased odds of being labeled with a high aggression risk behavioral health flag compared with White patients.

There is recent evidence that Black and Hispanic children are using the ED for mental health–related concerns at rates growing faster than those for White children.^4,5^ Accumulating evidence also indicates that both explicit and implicit bias among clinicians contribute to inequities in health care delivery^17,18,19,20,21,22^ for youths in the ED. In a study conducted by Johnson and colleagues,^21^ cognitive stressors in the ED were associated with increased implicit bias, and most physicians in the study exhibited implicit pro-White and anti-Black bias with respect to children. Black youths experience racial biases in the perception of their age, anger, and threat level. Black pediatric patients are more likely to be brought to the ED by police for agitation,^23^ are more likely to be subject to adultification bias^24,25^ and anger bias,^8^ and are more commonly associated with perceived threat compared with White pediatric patients.^26^ These racial biases can result in “unfair expectations of behaviors and harsher consequences for misbehavior.”^27^

Our findings that Black youths had higher odds of being labeled with a high aggression risk behavioral health flag despite having responses similar to those of White youths to the aggression risk evaluation tool could be suggestive of the racial bias that Black youths are more aggressive, leading to differential placement of high aggression risk flags. This finding also aligns with studies on racial differences in the treatment of mental health–related concerns conducted in adult EDs.^14,28^ These studies found that Black patients were more likely to have behavioral health flags in their EHR, warning staff that they were at risk of violence.^14,28,29^ In addition, Agarwal et al^14^ reported that Black patients with a behavioral health flag experience differences in ED clinical care outcomes, such as longer wait times and being less likely to undergo imaging or laboratory testing, compared with White patients, whereas Robinson and colleagues^29^ showed that having a flag for history of violent behavior increased odds of physical restraint use in an urban ED.

The differential application of behavioral health flags has the potential to transmit bias into the EHR and reinforce the existing structural racism that contributes to the overall health, safety, and well-being of minoritized youths.^4^ Systemic racism affects health care access, with minoritized youths accessing EDs for mental health care at rates increasing faster than those for their White peers.^4^ An aggression risk behavioral health flag could be reinforcing negative perceptions about patients and contributing to bias. The bias introduced by the behavioral health flag could lead to differences in ED clinical care outcomes and health disparities, which could unintentionally perpetuate a cycle of racism, prejudice, bias, and differential treatment. Given our findings in the context of accumulating evidence highlighting inequities in mental health care, focus on addressing potential biases and their impact on health care provision and outcomes is critically needed. Future studies should investigate the underlying factors, both structural and interpersonal, that may contribute to racial and ethnic differences in being labeled with a high aggression risk behavioral health flag, as well as further elucidating the long-term implications and unintended consequences of this flag on patient care. A structural approach could be to use a validated aggression risk screening tool with standardized scoring to help mitigate transmitting negative stereotypes about minoritized patients through the EHR-embedded behavioral health flags. The current evaluation tool does not have a standard scoring process to inform the placement of aggression risk behavioral health flags, and our findings show that such flags are being applied subjectively. Previous work has outlined the benefits and drawbacks to different types of violence or aggression screening tools.^30^ However, even standardized aggression screening tools could be flawed and, thus, should have ongoing evaluation for inequitable application. Alternative efforts to prevent aggression, such as environmental changes like reducing police presence, de-escalation training, trauma-informed interventions, and individualized behavioral health plans for youth aggression, could also help reduce racial bias in the ED.^31^ A study by Gonzales et al^32^ found that adult patients had varying perspectives on the use of behavioral health flags in the ED. Some patients believed that the use of a flag could mitigate violence and personalize care, whereas others were concerned of potential harms of the flag including negatively impacting care. More studies are needed to help understand the pediatric patient and family perspective, and to investigate how being labeled with a high aggression risk behavioral health flag can impact the patient experience and clinician decision-making.

### Limitations

There are several potential limitations to this study. First, we used EHR documentation of race and ethnicity. Although standard practice is to obtain self-reported data, it is possible that race and ethnicity are documented by the registration staff according to perception rather than self-report. However, when evaluating the impact of patient race and ethnicity on the placement of behavioral health flags, perceived race and ethnicity may be more informative. Second, because this study used data from the EHR, we are unable to determine whether there were other clinical factors aside from the screening questions that played a role in the assignment of the aggression flags. Third, our results are based on a single pediatric ED in an urban area and may not be generalizable to all pediatric EDs more broadly.

## Conclusions

In conclusion, we identified racial and ethnic differences in the use of an aggression risk behavioral health flag in the pediatric ED, with Black youths being labeled with a high aggression risk behavioral health flag more often than White youths, even when similar responses were documented in an aggression risk evaluation tool. These findings suggest that Black youths are discriminately being flagged as at high aggression risk. More work is needed to identify the reasons for this difference and to better understand the benefits and unintended consequences of behavioral health flags on patient care.

## Notes

Supplement.
Data Sharing Statement
