# Supplementary material for: Behavioral Health Flag Use by Race and Ethnicity in a Pediatric Emergency Department
Source: JAMA Netw Open. 2025 May 9;8(5):e259502. doi: 10.1001/jamanetworkopen.2025.9502 (PMC12065025; doi:10.1001/jamanetworkopen.2025.9502)
Supplement: Supplement. — Data Sharing Statement [file jamanetwopen-e259502-s001.pdf]

Data Sharing Statement

Foltz. Behavioral Health Flag Use by Race and Ethnicity in a Pediatric Emergency Department.
JAMA Netw Open. Published May 09, 2025. doi:10.1001/jamanetworkopen.2025.9502

Data
Data available: No
